# Maternal Dietary Carbohydrate and Pregnancy Outcomes: Quality over Quantity

**DOI:** 10.3390/nu16142269

**Published:** 2024-07-14

**Authors:** Lamei Xue, Xiaofang Chen, Juan Sun, Mingcong Fan, Haifeng Qian, Yan Li, Li Wang

**Affiliations:** 1 State Key Laboratory of Food Science and Technology, School of Food Science and Technology, Jiangnan University, Wuxi 214122, China; 6170112095@stu.jiangnan.edu.cn (L.X.); 18751554005@163.com (J.S.); fanmingcong@yeah.net (M.F.); qianhaifeng@jiangnan.edu.cn (H.Q.); 2 School of Public Health, Shanghai Jiao Tong University School of Medicine, Shanghai 200025, China; 184656@shsmu.edu.cn

**Keywords:** carbohydrates, pregnancy, glycemic index, offspring, metabolic syndrome

## Abstract

Dietary nutrition plays a crucial role in determining pregnancy outcomes, with poor diet being a major contributor to pregnancy metabolic syndrome and metabolic disorders in offspring. While carbohydrates are essential for fetal development, the excessive consumption of low-quality carbohydrates can increase the risk of pregnancy complications and have lasting negative effects on offspring development. Recent studies not only highlighted the link between carbohydrate intake during pregnancy, maternal health, and offspring well-being, but also suggested that the quality of carbohydrate foods consumed is more critical. This article reviews the impacts of low-carbohydrate and high-carbohydrate diets on pregnancy complications and offspring health, introduces the varied physiological effects of different types of carbohydrate consumption during pregnancy, and emphasizes the importance of both the quantity and quality of carbohydrates in nutritional interventions during pregnancy. These findings may offer valuable insights for guiding dietary interventions during pregnancy and shaping the future development of carbohydrate-rich foods.

## 1. Introduction

The rising prevalence of chronic metabolic diseases like obesity and type 2 diabetes (T2DM) is increasingly impacting younger populations. Alongside the lifestyle of children and adolescents themselves, maternal stress, diet, rest, environment, and other factors during pregnancy are significant contributors to the heightened risk of metabolic diseases in offspring [1]. The nutrients consumed by pregnant women serve as energy sources for both maternal energy expenditure and fetal growth and development [2]. Dietary nutrition plays a crucial role in shaping pregnancy outcomes, influencing not only the metabolic health of pregnant women and fetal environmental exposure in utero, but also indirectly impacting fetal growth and development by altering maternal metabolic characteristics. These modifications can have enduring effects on the metabolic health of offspring postnatally [3]. Therefore, providing scientific guidance to pregnant women on healthy eating habits and lifestyle can help mitigate the global burden of chronic metabolic diseases.

Pregnant women should increase their energy intake during pregnancy, with carbohydrates accounting for about half of their total energy intake. This increase is essential to meet the brain glucose needs of both the mother and fetus [2]. However, there has been a decline in carbohydrate intake among some populations since 2005, leading to many pregnant women consuming less than the recommended dietary allowance [4,5]. Some scholars argue that it may be necessary to reassess the required carbohydrate amounts for pregnant women and potentially increase the intake based on the current understanding [6]. This is because, in addition to the significant glucose needs of the mother’s and fetal brain, the placenta also relies on glucose as its primary energy source and is as reliant on maternal glucose as the brain [6]. Thus, as a key source of energy during pregnancy, carbohydrate food consumption should meet the requirements necessary for the normal growth and development of pregnant women and fetuses.

Carbohydrates, as the primary source of energy, play a crucial role in elevating blood sugar levels after meals compared to other macronutrients [7]. Pregnancy is a special physiological period characterized by gradual changes in basic glucose metabolism and postprandial glucose metabolism, leading to decline in insulin sensitivity and abnormal glucose metabolism [8]. Typically, insulin sensitivity decreases by 50% to 60% in the second trimester when compared to pre-pregnancy levels [9]. Overweight pregnant women or those with a history of type 2 diabetes are generally advised to restrict carbohydrate consumption during pregnancy [10]. Furthermore, fluctuations in blood sugar levels due to carbohydrate intake in pregnant women are linked to fetal birth weight and brain neurodevelopment. It is important to recognize that there is a wide range of carbohydrate-rich foods available, including whole foods and complex carbohydrates. High-quality carbohydrates elicit a different energy metabolism process compared to low-quality carbohydrates [7,11,12].

This article provides an overview of the impact of maternal carbohydrate intake on the health of both mothers and offspring. It emphasizes the significance of consuming adequate and high-quality carbohydrates during pregnancy. This study offers a theoretical foundation for enhancing pregnancy outcomes through dietary intervention.

## 2. Carbohydrates and the Role of Carbohydrates in Pregnancy

### 2.1. Carbohydrate Quality

#### 2.1.1. Glycemic Index and Glycemic Load of Carbohydrates

More than 55% of the carbohydrates in diet are associated with elevated postprandial plasma glucose (PPG). When foods containing carbohydrates are ingested, the resulting change in PPG is a glycemic response (GR). The glycemic response induced by a portion of food containing 50 g of available carbohydrates is the glycemic index (GI). Glycemic load (GL) is a measure of the quality and quantity of the carbohydrate. “Available carbohydrates” refers to the amount of carbohydrates that have been digested, absorbed, and metabolized. GI is used not only to classify and compare individual foods but also extended to mixed and whole diets. Foods with a GI above 70 on the glucose scale are considered high GI foods, indicating rapid digestion, absorption, and metabolism of carbohydrates [13]. Conversely, low GI foods have a GI of less than 55, leading to a slower rise in blood sugar [14]. For example, rice, refined white flour, and potatoes can cause rapid spikes in blood sugar levels, while whole grains, vegetables, and certain fruits can result in lower postprandial blood sugar levels. Different carbohydrate subtypes, which indicate quality, have varying rates of digestion and absorption, potentially resulting in different impacts on postprandial blood sugar levels [15]. There is no specific numerical boundary between low-quality markers are described, and these are usually divided into four main markers: higher dietary fiber, lower sugar intake, low GI and GL, and food sources of carbohydrate (whole grain, pulses, fruit) [16].

Maternal glucose response is influenced not only by the total amount of carbohydrate, but also by the type of carbohydrate consumed [17]. Slow-digesting, absorbing, and metabolizing carbohydrates are typically high in dietary fiber and rich in phytochemicals [18]. In their evaluation of carbohydrate quality, Reynolds et al. analyzed a large dataset of 135 million person-years from prospective studies and 58 clinical trials involving 4635 adult participants. They concluded that the relationship between carbohydrate quality and health outcomes, as assessed by dietary fiber, is of moderate certainty, while whole grains show a low-to-moderate level of evidence [19]. 

Carbohydrates are derived from various sources, each with distinct physiological functions. Therefore, the assessment of carbohydrates should consider the specific type or source. Typically, simple carbohydrates result in greater postprandial blood glucose spikes compared to complex carbohydrates. Numerous countries advocate for a higher consumption of complex carbohydrates. Research, as outlined in Table 1, indicates that various commonly consumed carbohydrates have unique impacts on maternal health during pregnancy.

#### 2.1.2. Simple Carbohydrates (Sugars)

The types of carbohydrates are also classified according to their chemical structure: simple carbohydrates usually include monosaccharides and disaccharides. Monosaccharides include glucose and fructose, which have only one aldehyde or ketone unit, and disaccharides can be hydrolyzed into two monosaccharides. The glucose and fructose in fruits, the large amount of galactose in milk, cream and beets, the sucrose in white and brown sugar, the lactose in milk powder and cheese, and the maltose in sugarcane and malt are all simple carbohydrates also called simple sugars.

In addition to being found in whole foods, the other major source of simple sugars that people consume in their daily diet is added sugars. Added sugars is another important category of sugar, which refers to all monosaccharides and disaccharides used in processed foods and beverages, as well as sugars added to foods that do not naturally occur [33,34]. It does not include natural sugars found in vegetables, fruits, and milk. Sugar-sweetened beverages are the largest source of added sugar consumption, including carbonated beverages, non-carbonated beverages, fruit drinks, sports drinks, and energy drinks. All monosaccharides and disaccharides added to foods, as well as sugars found naturally in honey, syrups, and fruit juices, are also known as free sugars. 

Excessive sugar intake has been linked to an increased risk of various chronic metabolic diseases, including obesity, cardiovascular disease (CVD), T2DM, metabolic syndrome, and non-alcoholic fatty liver disease (NAFLD) [35]. Recent surveys indicate a decline in consumption of sugar-sweetened beverages in developed countries, while their consumption is on the rise in many developing nations [36]. Both natural fruit juices and sweetened beverages contain similar amounts of free sugar. Although the superiority of natural fruit juices over sweetened beverages is still debated, some studies suggest that natural fruit juices may be associated with a lower risk of non-alcoholic steatohepatitis [37], inflammatory bowel disease [38], and Alzheimer’s disease [39] when compared to sugar-sweetened and artificially sweetened beverages. Nevertheless, further randomized controlled trials are necessary to compare the metabolic effects of fruit juices and sugary drinks in order to establish accurate public health guidelines regarding the types and quantities of free sugars in our diets, ultimately aiding in the prevention of obesity and related health issues.

#### 2.1.3. Complex Carbohydrates (Starches)

Complex carbohydrates are either oligosaccharides containing up to 10 monosaccharide units, or polysaccharides containing very long monosaccharide chains. The starch contained in noodles, bread, etc., is a polysaccharide, which is digested and decomposed into glucose by amylase after intake, and then slowly enters the blood without causing a sharp rise in blood sugar. Foods containing polysaccharides mainly include grains, legumes, vegetables, etc., and these natural whole foods are rich sources of high-quality complex carbohydrates.

A high intake of dietary fiber and whole grain foods is more clearly associated with good health outcomes, but this is not just attributed to GI or GL. While the GI provides a measure of the glycemic potential of the carbohydrate content of a food, some foods with a low GI may have other detrimental properties, such as many confectionery products containing added fructose or sucrose and complex foods containing both saturated fat and carbohydrates [40]. Foods that contain fiber should be chewed before passing through the stomach and into the small intestine, as they affect satiety, glucose and insulin response, and lipid absorption. While recent systematic reviews have shown only small effects on appetite, satiety, or blood lipids, most of these studies have been conducted with specific fiber supplements, not whole foods [41,42,43].

Compared with refined grains, whole grains, in addition to retaining most of the dietary fiber, more importantly, are not deeply processed, retaining the characteristics of whole foods. More than 100 years of research have demonstrated that eating whole grains can effectively increase dietary fiber intake and reduce the risk of non-communicable diseases [44]. Vegetables and fruits are also important sources of dietary fiber intake, and studies reported that eating 200 g of fruits and vegetables per day reduces the risk of death from coronary heart disease, stroke by about 10%, and cardiovascular disease and cancer by smaller, but still significant amounts [45]. 

A meta-analysis of a series of systematic reviews and prospective studies on the association between carbohydrate quality and non-communicable disease incidence (such as heart disease, stroke, diabetes, cancers, and chronic respiratory diseases [46]), mortality, and risk factors published in 2018 and previous databases conducted by Reynolds et al. found that diets with a low GI or low glycemic load had a similar reduction in type 2 diabetes risk as whole grain foods [19]. However, the lack of support for the effects of a low GI or low GL diet on hemoglobin A_1C_ or blood cholesterol has been reported in many short-term (4–6 weeks) and medium-term (8–10 weeks) dietary intervention trials [47,48]. These studies demonstrate that consuming complex carbohydrates is not only advantageous for maintaining the health of individuals without chronic metabolic diseases, but also serves as a suitable nutritional treatment for those with such conditions. 

### 2.2. Carbohydrate Quantity

With the improvement in living standards, people are increasingly seeking not only the nutritional value of food but also higher sensory quality. Pregnant women following a low-carb diet often compensate for the lack of carbohydrate foods by increasing their intake of other items. This practice typically results in two negative effects. Firstly, they tend to opt for non-carb foods that mimic the sweetness and flavor of sugars found in carbohydrate-rich foods, while being low in calories [49]. Non-nutritive sweeteners fulfill these criteria. These sweeteners are progressively replacing free sugars in various processed foods, prompting some pregnant women to intentionally choose such products. Secondly, when pregnant women consume more fatty foods to make up for the caloric deficit from carbohydrates, the liver breaks down fatty acids, leading to the production of ketone bodies in the absence of glucose [50]. 

#### 2.2.1. Sugar Reduction and the Popularity of Non-Nutritive Sweeteners

Non-nutritive sweeteners (NNS) are zero- or low-calorie alternatives that have a high sweet intensity and potency compared to nutritive sweeteners which are mostly sugar alcohols [51]. The U.S. Food and Drug Administration (FDA) currently approves synthetic high-intensity sweeteners such as saccharin, aspartame, acesulfame-K, sucralose, neotame, and advantame. Additionally, natural high-intensity sweeteners include steviol glycosides, thaumatin, and *Siraitia grosvenorii* [52]. Various non-nutritional sweeteners exhibit different absorption characteristics and glycemic indices. Approximately 20–30 percent of sucralose is absorbed into the bloodstream, with the remaining 80–90 percent being excreted in feces and urine [53]. Acesulfame K and saccharin are not metabolized or stored by the body [54]. Aspartame undergoes complete digestion by esterases and peptidases in the gastrointestinal tract, yielding secondary metabolites that are then absorbed into the bloodstream [55]. Steviosides are not metabolized by human enzymes [56], but are metabolized by gut bacteria [57].

Non-nutritive sweeteners, although not sugar, are commonly found in a variety of foods, especially those that have a growing share of the beverage market. Therefore, we will also discuss the health effects of this ideal substitute for sugar-based substances on pregnancy. Non-nutritive sweeteners are sweet substitutes that were originally developed as an alternative to white sugar in tabletop packaging and diet drinks, but now they are widely used in thousands of foods and beverages. The Academy of Nutrition and Dietetics affirms the safety of consuming NNS during pregnancy and childhood, whereas the US Institute of Medicine and the American College of Obstetricians and Gynecologists do not provide specific guidance on NNS consumption during pregnancy [58]. 

In order to limit the intake of added or free sugars, many pregnant women choose sugar-free foods or beverages containing non-nutritive sweeteners, and there is an increasing number of foods with non-nutritive sweeteners on the market [49]. Thirty percent of pregnant women intentionally consume foods with added non-nutritive sweeteners [59]. Other women may make a conscious decision to consume non-nutritive sweeteners in an attempt to reduce gestational weight gain (GWG), or while undergoing medical nutritional treatment for pre-pregnancy or gestational diabetes [54]. Research indicates mixed findings regarding the impact of saccharin and sucralose on body weight and weight gain in pregnancy and lactation [60]. While some studies suggest a reduction in body weight, others show no significant effect on maternal weight [61]. The long-term health risks to mothers from NNS exposure during pregnancy remain inconclusive, with limited exploration in this area of research.

Some non-nutritive sweeteners, such as aspartame, can be fully broken down, while others circulate in the body without being metabolized, and can be detected in the blood, urine, and feces [53,55,62,63]. These sweeteners are primarily absorbed in the small intestine, can cross the placenta to reach to the fetus, and be transferred to the infant through breast milk [64,65]. The Academy of Nutrition and Dietetics asserts that non-nutritive sweeteners are safe to consume during pregnancy and childhood [54]. However, the American Institute of Medicine and the American College of Obstetricians and Gynecologists have not issued any specific guidelines on the consumption of non-nutritive sweeteners during pregnancy [54]. Clinical studies on non-pregnant individuals and animal models have shown that non-nutritive sweeteners can impact glucose absorption, appetite, insulin secretion, and fat production. Additionally, the consumption of these sweeteners may disrupt the gut bacterial microbiota, leading to various metabolic changes [54].

#### 2.2.2. Ketone Physiology during Pregnancy

In cases of glucose deficiency, the liver mitochondria produce ketone bodies by breaking down fatty acids. These ketones, which include acetone, 3-beta-hydroxybutyrate (3BHB), and acetoacetate (AcAc), are energy-rich metabolites. Healthy adults typically have ketone body concentrations ranging from 100 to 250 μM, while prolonged fasting or carbohydrate restriction can elevate these levels to 1 mM or even 20 mM [66]. When glucose supply is low or during starvation, fatty acids serve as an alternative energy source and are converted into ketone bodies. It is important to clarify that low-carb or ketogenic diets are typically defined based on the total amount of carbohydrates consumed daily, rather than as a percentage of total energy intake [67]. A low-carb diet results in insufficient glucose for promoting ketone production, leading to elevated ketone levels throughout the body. This altered metabolic state in the mother can impact both herself and the fetus [68].

If the carbohydrate intake in the diet is sufficiently low, the ketogenic effect will increase [69]. In non-pregnant individuals, consuming less than 50 g of carbohydrates per day is believed to enhance the ketogenic effects [67]. During pregnancy, ketogenic effects are intensified [70], but the precise minimum carbohydrate intake required to prevent an increase in these effects remains unknown. During the third trimester of pregnancy, levels of serum BHB and acetoacetic acid are found to be three times higher compared to non-pregnant women, indicating a state of accelerated starvation [70]. This increase in ketogenesis in pregnant women is attributed to various metabolic changes [68]. Placental growth hormone secretion in the second trimester antagonizes insulin function, leading to insulin resistance, increased lipolysis, and subsequently elevated ketogenesis [62]. Moreover, lower fasting blood sugar levels in pregnant women contribute to enhanced lipolysis and increased ketogenesis [71,72]. Numerous animal studies have indicated that heightened ketone concentrations during pregnancy can be detrimental to both the mother and fetus.

## 3. Dietary Carbohydrate and Pregnancy Complications

As one of the main dietary components, carbohydrate plays a complex and critical role in maintaining glucose and lipid metabolism homeostasis in the body. During pregnancy, glucose is transmitted to the fetus through the placenta, leading to increased fetal insulin secretion and the subsequent stimulation of fetal growth. It is important for pregnant women to achieve appropriate weight gain to support fetal growth and development. The recommended GWG for women with gestational diabetes is the same as that for normal pregnant women. A minimum intake of 175 g of carbohydrates per day is necessary to ensure adequate nutrient supply [73]. The fluctuation of blood glucose level and insulin sensitivity during pregnancy are important indicators of metabolic risk assessment in pregnant women [74]. Current evidence does not suggest that women with hyperglycemia or hyperinsulinism have different energy requirements compared to those with normoglycemia. Additionally, there is no specific recommendation for optimal calorie intake for women with gestational diabetes mellitus (GDM) [75]. International consensus on the daily intake of moderate carbohydrates for pregnant women has not been established.

Compared to other macronutrients, carbohydrates have a greater impact on raising blood sugar levels after a meal. Postprandial hyperglycemia mainly depends on carbohydrate intake, as eating large amounts of carbohydrates in one meal can lead to high blood sugar. Carbohydrates are the most important macronutrient for both healthy and women with GDM. In the short term, maternal hyperglycemia is associated with an increased risk of adverse pregnancy outcomes and increased risks of childhood obesity and type 2 diabetes in offspring. In certain regions, a low-carbohydrate diet is still the conventional treatment. For women who are obese, or who have achieved recommended GWG, a calorie restriction of 30–33% may be advisable. 

Since 2002, pregnant women have been advised to adjust their dietary energy intake according to their pre-pregnancy BMI on a trimester basis to prevent or reduce excessive gestational weight gain (Institute of Medicine, 2002) [76,77]. During the first trimester of pregnancy, no additional calories are needed, while an extra 340 kcal/day and 452 kcal/day are recommended during the second and third trimesters, respectively. It is recommended to increase calorie intake by 150, 200, and 300 kcal/day during the first, second, and third trimester for underweight women. For normal weight women, the suggested increase is of 0, 350, and 500 kcal/day, while for obese women it is of 0, 450, and 350 kcal/day [77,78]. While the recommended carbohydrate intake during pregnancy varies by region or country, in the United States, pregnant women are recommended to consume 175 g of carbohydrates per day, 45 g more than non-pregnant women, to ensure that the fetus receives enough energy in the form of glucose [50,76,79]. According to the Chinese Nutrition Society, it is recommended that the energy intake during the first trimester of pregnancy should not increase, while in the second and third trimester of pregnancy, the energy requirements of pregnant women should be increased by 300 kcal and 450 kcal per day on the basis of the energy requirements of non-pregnant women, respectively. Additionally, pregnant women are advised to consume an average of 130 g carbohydrates daily, which should account for 50–60% of their total daily energy intake.

It is not feasible to solely restrict carbohydrate intake for pregnant women. Severe restriction in carbohydrates may lead to replacing carbohydrate energy with fat, especially with the abundance of processed foods and prevailing trend of over-nutrition [10]. Carbohydrates play a crucial role in the nutrition of pregnant women and fetal development. A randomized control trial involving 800 women without diabetes, in their second pregnancy with a history of delivering an infant weighing over 4 kg, found that following a low-glycemic index diet during pregnancy led to lower GWG and reduced maternal glucose intolerance [80]. By adjusting the types of carbohydrate-rich foods and increasing the consumption of high-quality carbohydrates, it is possible to prevent ketonemia and ketonuria resulting from excessive fat intake due to adequate carbohydrate consumption, which can negatively impact fetal mental or motor function. Therefore, during pregnancy, carbohydrates should mainly come from low-glycemic starches and naturally fiber-rich foods like vegetables, legumes, fruits, and whole grains, while limiting the intake of added sugars and refined carbohydrates [81].

### 3.1. Low-Carbohydrate Diets and maternal health 

Pregnant women who are obese or have a history of T2DM are advised to limit carbohydrate intake during and after pregnancy as a key component of medical nutrition treatment. The Endocrine Society Clinical Practice guidelines recommend that women with abnormal sugar metabolism or impaired insulin sensitivity should limit carbohydrate intake to 35–45% of total calories [82]. Globally, the nutritional recommendation for GDM is to limit carbohydrates [83]. A non-randomized study found that carbohydrate restriction improved blood sugar control in patients with GDM and reduced the need for insulin therapy after 6 weeks of treatment with a carbohydrate-restricted diet [84]. 

Numerous studies suggest that low-carbohydrate diets (LCDs) may have benefits during pregnancy, including lowering blood sugar, improving insulin resistance, and reducing the risk of gestational diabetes. However, conflicting research exists, indicating that restricting dietary carbohydrates could lead to higher fat intake, particularly in obese pregnant women who have easy access to processed foods [10]. A prospective cohort study involving 722 incident cases of T2DM reported that a low-carbohydrate dietary pattern with high protein and fat intake, primarily from animal-source foods was linked to a higher risk of T2DM. Conversely, diets high in plant-based fats and proteins were not significantly associated with this risk [20]. Additionally, in a cross-sectional study, 614 pregnant women in Singapore were recruited to evaluate maternal diet quality using the Health Eating Index in Asian Pregnant women (HEI-AP). Rayner et al. conducted a dietary questionnaire on a sample of 9689 Australian women, revealing a significant association between carbohydrate restriction and an increase in risk of type 2 diabetes in middle-aged women. This relationship was observed to be independent of a history of GDM [21]. The results indicate that pregnant women consuming a low-quality diet with high fat intake and low protein and carbohydrate intake during pregnancy had wider retinal vein diameters, suggesting suboptimal microvasculature [85]. 

There are various hypotheses regarding the biological mechanism underlying the connection between a low-carbohydrate diet and postprandial glucose levels. One perspective suggests that restricting carbohydrates may lead to a reduction in the consumption of essential food components like whole grains, vegetables, and fruits, resulting in inadequate dietary fiber intake and potential health issues. Conversely, pregnant women may need to increase their consumption of high-protein and high-fat foods to compensate for the lack of carbohydrates [86]. Maternal increased fat intake during pregnancy was found to be related to the development of glucose abnormalities [23]. 

However, the safety of such diets lacks confirmation from clinical and pathological studies. Animals research has demonstrated that ketogenic diets can induce glucose intolerance, hepatic endoplasmic reticulum stress, steatosis, cell damage, and macrophage accumulation in mice, while not impairing insulin-induced Akt phosphorylation in the liver or systemic insulin reactivity [87]. Furthermore, diets rich in animal protein have been associated with elevated plasma levels of branched-chain amino acids, which have been linked to insulin resistance and diabetes in metabolomics studies [88]. Additionally, high-fat diets have been shown to negatively impact glucose tolerance, potentially due to alterations in fatty acid composition from dietary fat modification, particularly animal fats, affecting insulin binding and intestinal health [89]. 

Short-term low-carb diets have demonstrated an improvement in blood sugar control for individuals with gestational diabetes mellitus (GDM) [90,91]. It is crucial to recognize that a prolonged carbohydrate-restricted diet may lead to complications like malnutrition, ketoacidosis, and ketonuria in pregnant women with GDM or metabolic issues like obesity and hypertensive disorders of pregnancy (HDP) [20,86,92]. The definition of harmful ketone levels during pregnancy remains unclear. A study involving 180 pregnant women with gestational diabetes mellitus (GDM) revealed that approximately 1.6% exhibited high serum ketone levels, while 5% had moderate levels [93]. Various studies have reported differing findings on high urinary ketones in pregnant women, with prevalence ranging from 9% to 89% [90,94,95]. Most research on urinary ketones in pregnant women has focused on those with diabetes, leading to wide variations in reported prevalence. Despite the general consensus that ketogenesis increases during pregnancy, the exact prevalence of urine ketone testing remains unknown.

This study found no significant association between artificially sweetened beverages intake and measures of metabolic disease in women with a history of GDM. Women who regularly consumed artificially sweetened beverages during pregnancy showed higher levels of glycated hemoglobin, insulin, triglycerides, and obesity. Upon adjusting for covariates, the association between artificially sweetened beverages intake and these outcomes disappeared [27]. One study reported an increased consumption of non-nutritive sweeteners during pregnancy compared to pre-conception [96]. Although there are currently no specific guidelines for the consumption of non-nutritive sweeteners for pregnant women, we do not recommend a high consumption of non-nutritive sweetener foods or beverages based on the results of some experiments in non-pregnant people and animal models. In a word, pregnant women typically avoid consuming excessive amounts of carbohydrates and intentionally reduce their intake of artificially sweetened beverages and other low-quality carbohydrates. However, it is not advisable to prioritize NNSs as a means of reducing calorie intake.

Taken together, during pregnancy, it is important to adjust the diet to account for the increased energy expenditure while aiming for appropriate gestational weight gain. The maternal brain and the developing fetal brain rely on glucose as their primary fuel source. To support normal fetal growth and brain development, it is advised to consume an additional 35 g of carbohydrates per day during pregnancy, as recommended by the Institute of Medicine (IOM).

### 3.2. High-Carbohydrate Diets and maternal health

#### 3.2.1. Low-Quality Food Sources of Carbohydrate and Maternal Health

It should be emphasized that excess weight during pregnancy is associated with many common adverse effects of pregnancy. A large increase in carbohydrate intake can lead to significant weight gain, especially refined carbohydrates and processed sugars which can make the body fatter. Excessive nutrient intake or excessive sugar intake increase plasma insulin concentrations and promote lipid storage in adipose tissue. Renault et al. also reported that the sugary foods most strongly associated with GWG were sweets, snacks, cakes, and soft drinks. Women who consumed sweets twice a day or more had an average gestational weight gain that was 5.4 kg greater than women who consumed sweets less than once a week [24]. Along these lines, greater adherence to a diet rich in added sugar, processed snacks [25,97], and refined grains [97] was found to be detrimental to pregnancy outcomes and even less beneficial to high blood pressure risk.

About 92% of women are aware that consuming too much sugar increases the risk of diabetes and cardiovascular disease [98]. A prospective cohort study of dietary carbohydrate quality in 9317 Chinese women, as assessed by food frequency questionnaires during pre-pregnancy, first trimester, and second trimester, revealed that pregnant women had highest scores of dietary GI during these periods exhibited a 12%, 25%, and 29% higher risk of developing GDM compared to those with the lowest scores [26]. 

#### 3.2.2. High-Quality Food Sources of Carbohydrate and Maternal Health

Diets rich in high-quality carbohydrates may lead to higher GWG, but this does not necessarily result in adverse outcomes such as increased blood sugar levels and obesity in offspring. Several studies have suggested that increasing the consumption of traditional dietary patterns rich in whole grains, legumes, vegetables, and lean meats while reducing the intake of highly processed foods high in sugar, fat, and additives may be beneficial [31]. Hillesund et al. conducted an investigation with 66,597 women from the Norwegian Mother and Child Cohort Study. They found that women with higher adherence to the New Nordic Diet, which mainly consists of foods such as fruits and vegetables, whole grains, potatoes, fish, game, milk, and drinking water, were associated with a reduced risk of excessive GWG [28]. The rationale behind recommending a diet abundant in high-quality carbohydrates, such as whole grains, for pregnant women is to ensure adequate nutrition for fetal growth without causing excessive maternal weight gain or abnormal weight loss [99]. 

Australian researchers imparted 33 isocaloric diets on 700 mice and found that a high-carbohydrate diet (70%) is beneficial to metabolic health, and it is worth mentioning that the high content of carbohydrates has a certain resistant starch [100]. Population studies on the effects of diets high in quality carbohydrates, such as low GI whole grains, on the health of pregnant women have shown positive results. A diet consisting of low-GI (LGI) foods such as whole grains is effective in preventing GDM complications by normalizing fasting blood glucose and postprandial blood glucose [29]. Hu et al. [30] conducted a randomized controlled trial of 140 women with GDM who were randomly assigned to an LGI diet or a normal diabetes control diet. In the LGI diet group, refined grains in main meals were replaced by LGI foods. It was found that the LGI group had significantly lower postprandial blood glucose levels than the control group, although the total energy and carbohydrate content were similar between the two groups. In post-GDM women, lowering the GI of a healthy diet significantly improved glucose tolerance and weight loss compared with a traditional low-fat diet with a similar energy prescription [101]. 

In addition to the proportion of macronutrients in the diet affecting health during pregnancy, other nutritional factors, dietary habits, and lifestyle factors are also key influencing factors. Therefore, dietary patterns that rely on chronic carbohydrate deficiency need to be treated with caution by every pregnant woman. In a randomized crossover study, pregnant women were randomly assigned to a high-carbohydrate diet (60%) or a low-carbohydrate diet (40%), whereas fasting blood glucose in pregnant women on a higher-carbohydrate diet was similar in both groups (15–20%) [102]. Instead, low-carb diets lead to higher fasting blood sugars. The reason for this is that low-carbohydrate diets are higher in fat, and the higher fatty acids from fat can lead to insulin resistance in pregnant women, which can lead to increased fasting blood sugar [32]. In conclusion, the findings suggest that a dietary pattern consisting primarily of high-quality carbohydrates, protein, and lipids is beneficial for long-term weight regulation in pregnant and postpartum women.

## 4. Dietary Carbohydrate and Offspring Health

**Figure 1:**
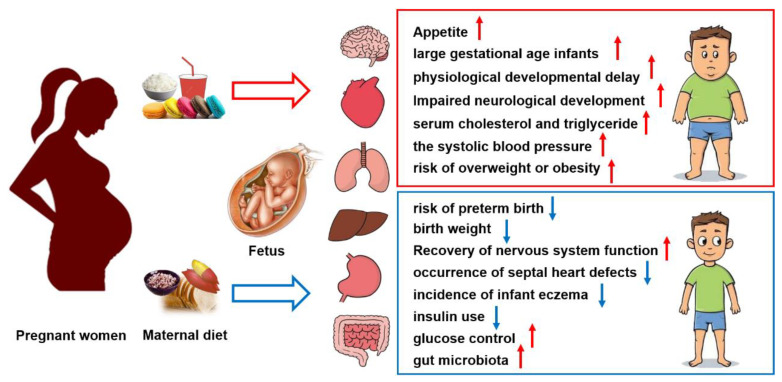
The quality of carbohydrates consumed during pregnancy affects the health of the offspring. Red arrows indicate an increase in the corresponding level, risk, or abundance. Blue arrows indicate a decrease in the level, risk or function used.

There is growing evidence suggesting that the mother’s dietary intake during pregnancy is closely related to the developmental programming of the fetus. As summarized in Table 2, improper macronutrients in the mother’s diet can lead to high blood sugar during fetal development and are associated with an increased prevalence of T2DM in adulthood [103,104]. North et al. conducted a systematic review of randomized controlled trials including 14 articles from 11 RCTS involving 3614 participants [105]. Their findings indicated that maternal diet did not significantly impact insulin, *C*-peptide, or glucose levels in newborns’ cord blood. However, infants born to mothers adhering to a low glycemic load (GL) diet exhibited reduced skin fold thickness. Furthermore, interventions offering personalized nutritional counseling to obese women were linked to lower obesity rates in their offspring. These results highlight the potential benefits of lifestyle-based dietary interventions in improving blood sugar levels and preventing excessive obesity [105]. As shown in Figure 1, high levels of saturated fat or refined sugar in the mother’s diet is one of the major factors in the development of obesity and metabolic syndrome in offspring. The impaired maternal metabolic status discussed above (GDM, HDP, GWG), which is caused by overnutrition or inappropriate nutritional status, affect fetal growth, birth weight, and the development of chronic metabolic diseases such as obesity in later generations.

### 4.1. Low-Carbohydrate Diets and offspring health

Mothers on very low-carbohydrate diets showed reduced fertility and litter size, an increased risk of fatal ketoacidosis during lactation, and stunted growth in their offspring. Research has shown that a maternal diet high in fat and low carbohydrates during pregnancy can lead to impaired glucose tolerance and insulin signaling in newborn piglets. This type of diet also decreases mitochondrial function, down-regulates the protein levels of slow muscle fibromyosin heavy chain I (MyHC I), and up-regulates the protein levels of fast muscle fibromyosin heavy chain IIb (MyHC IIb) and IIx (MyHC IIx) in soles [116]. Viana et al. conducted a systematic review and meta-analysis of randomized clinical trials (RCTs) of dietary intervention in GDM or hyperglycemic pregnancy and found that restricted diets such as low carbohydrate did not alter maternal and newborn outcomes, while low GI diets were associated with higher insulin sensitivity and lower birth weight in pregnant women [107].

The low-carbohydrate ketogenic diet during pregnancy has been reported to directly affect the brain of pregnant women and damage the neurodevelopment of offspring. However, pregnant women who discontinue this ketogenic diet in the early postpartum period may restore nervous system function by initiating compensatory processes. Kosiek et al. found significant weight loss and neurodevelopmental delays in the offspring of mothers who followed a ketogenic diet during pregnancy [106]. In addition, brain analysis of offspring exposed to very low-carbohydrate diets showed significant alterations in neonatal brain structure, leading to later behavioral changes [108]. Fetuses exposed to a low-carb/ketogenic diet in utero exhibited anxiety and depression in adulthood, with many neuroanatomical differences, and affecting their behavior as adults [117]. Maternal carbohydrate intake during pregnancy is crucial for meeting the needs of fetal growth and development. Specifically, fetal brain neurological development requires sufficient glucose as an energy supply. 

The role of maternal ketones in fetal central nervous system development remains unclear. By 8–10 weeks of gestation, the fetal brain expresses enzymes that break down ketones [118]. Elevated maternal ketones, as seen in situations like starvation and low sugar states, lead to the increased activity of these enzymes in the fetal brain [119]. Some studies suggest that ketones could be a significant source of fuel for fetal brain development, but the evidence is limited and definitive conclusions are challenging to make [118]. Two animal studies have explored maternal ketones and fetal brain ketone utilization. In vitro experiments have shown that ketone bodies acetoacetate and BHB can independently inhibit the neonatal biosynthesis of pyrimidine and purine in fetal rat brains, potentially linking maternal ketones to lower IQ in children [120,121].

The physiological response of the human fetal brain may differ from that of rodents, with different enzymes and pathways as well as differences in amino acid abundance or environmental pH, and it is possible that ketone bodies in the human fetal brain do not continue the de novo synthesis of purines and pyrimidines [122]. Whether in vitro studies in rodents mimic what happens in humans is unclear. A retrospective study conducted in the United States during the 1960s examined the relationship between prenatal ketones and children’s IQ [123]. The study found a link between maternal ketone levels in diabetic pregnant women and lower Stanford–Binet IQ scores in their offspring. Pregnant women were subjected to irregular urine testing, with high ketone levels resulting in classification as ketone-positive cases, while ketone-negative cases showed no ketones or only trace amounts in the urine. The children of these women were tested for IQ levels at age 4, revealing that the offspring of 111 ketone-positive women had notably lower IQ scores compared to the children of ketone-negative women [123]. Therefore, pregnant women following a low-carbohydrate diet pattern may not be beneficial for the health of their offspring.

The effects of non-nutritive sweetener intake during pregnancy on pregnancy outcomes and the long-term health of offspring remains inconclusive, and it is unclear how they affect the health of offspring later in life. Nettleton et al. found that, even if offspring did not directly consume low-calorie sweeteners, maternal consumption of low-calorie sweeteners such as aspartame/stevia in combination with a high-fat/high-sugar diet may adversely affect body weight regulation, glucose control, and gut microbiota in maternal mice and their offspring [114]. Similarly, low doses of aspartame and stevia during pregnancy in mice resulted in weight regulation and glucose control issues in both mothers and offspring, with early life gut microbiota dysbiosis observed in offspring despite not directly ingesting these sweeteners [114].

### 4.2. High-Carbohydrate Diets and offspring health

#### 4.2.1. Low-Quality Food Sources of Carbohydrate and Offspring Health

Clinical guidelines and public health policy recommendations for pregnant women’s diets should advise reducing the intake of free sugars to potentially lower the risk of allergies in offspring [124]. Research from the Avon Longitudinal Study of Parents and Children, with a sample size of 8956, analyzed the relationship between maternal free sugar consumption during pregnancy and the development of asthma, wheezing, atopy, and lung function in their offspring. The findings indicated a positive correlation between maternal free sugar consumption and asthma (*p*-trend = 0.004) as well as atopy (*p*-trend = 0.006) in children aged 7–9 years [125]. This finding underscores the importance of maternal diet on the future health of their children, as even if offspring maintain a healthy diet later in life, they still face an elevated risk of respiratory and atopic diseases. Limited studies indicate a link between high free sugar intake during pregnancy and offspring allergies [126]. Zhu et al. examined the relationship between refined grain consumption during pregnancy and offspring growth. They discovered that higher refined grain intake in mothers with GDM was significantly linked to increased birth weight in children and a higher likelihood of being overweight or obese by age 7 [111]. Furthermore, pregnant women on low-carb diets who replaced fat with 5% of energy from high-quality carbohydrates showed a reduced risk of IgE-mediated allergic disease [127]. 

A high-sugar, high-fat diet increased body weight, visceral fat, and serum total cholesterol levels in newborn rats, while decreasing hypothalamic weight. This diet also led to increased triglyceride and leptin levels as well as hypothalamic inflammation in adult male offspring [112]. On the other hand, a high-carbohydrate, low-protein diet in mothers reduced overall DNA methylation levels in the offspring’s liver. In addition, a high starch-to-fat ratio diet in sows and newborn piglets results in reduced plasma LDL cholesterol concentrations. Moreover, the high starch fat diet of sows increased the transcriptional abundance of fatty acid synthase in newborn piglets [128]. Lastly, offspring exhibited the highest systolic blood pressure in childhood when mothers had a dietary ratio of 16% protein and 40% carbohydrates [113].

#### 4.2.2. High-Quality Food Sources of Carbohydrate and Offspring Health

Higher maternal carbohydrate intake during the second trimester was found to be inversely associated with fat index, while higher carbohydrate energy intake in the third trimester was linked to a lower newborn fat index. This suggests that a high-carbohydrate low-fat diet during different stages of pregnancy could potentially reduce the obesity index of the newborn [129]. In a study by Hernandez et al., they challenged the traditional low-carbohydrate/high-fat diet by implementing a 7-week dietary intervention during late gestation for overweight or obese women with GDM. Furthermore, in most low-risk pregnant women, a healthy diet quality index before and during the first trimester was associated with the birth weight of the newborn [109,110]. Furthermore, clinical interventions targeting short-chain fatty acids (SCFAs) have shown promise in improving offspring health [130]. The results indicate that a diet rich in complex carbohydrates and low in fat not only improved the mother’s insulin resistance but also benefitted the offspring by reducing metabolic disorders and lowering the risk of obesity [131]. 

A large number of studies have provided conclusive evidence that maternal diet can regulate offspring neurocognitive function by modulation of SCFA levels in the gut. Recently, Liu et al. revealed that a high dietary fiber intake during pregnancy can mitigate synaptic damage and microglial maturation defects in offspring mice by restoring maternal gut microbiota, thereby reducing cognitive and social disorders stemming from maternal obesity [132]. Additionally, Sideratou et al. compared the risk of obesity in the offspring of female mice fed rapidly digested starch and slowly digested starch (other nutrients being the same). Through this mouse model, obesity-related genes were found to be regulated by different maternal diets in the placenta and the offspring’s hypothalamus, again demonstrating that a high-GI carbohydrate diet is associated with higher appetite in the offspring [115]. Martin et al. investigated the impact of two high-fat diets, equal in glycemic index but differing in carbohydrate digestibility, on offspring health. Their findings revealed that offspring of insulin-resistant rats fed a high-fat diet with rapid carbohydrate digestibility exhibited excessive adipogenesis [133]. 

Conversely, the dietary early programming associated with a slow-digesting carbohydrate diet demonstrated a synergistic and beneficial effect on various tissues including muscle, liver, and adipose tissue, thereby contributing to the maintenance of metabolic homeostasis in offspring [134]. Callanan et al. studied the influence of a low GI diet during pregnancy on offspring weight and concluded that such a diet did not affect the anthropometric outcomes of the offspring at age 5 [135]. The reasons for this study may be related to the time and cycle of dietary intervention during pregnancy, and the effects of prenatal nutrition on offspring are long-term, including resistance to chronic disease risk in later life, so no differences were observed in body measurements of phenotypes in early life. Furthermore, the intake of carbohydrates during pregnancy is intertwined with the consumption of other nutrients in the maternal diet [135].

## 5. Conclusions and Recommendations for Future Research

This review article delves into the importance of a moderate consumption of specific carbohydrate diets during pregnancy, emphasizing the impact of carbohydrate quality on maternal health, fetal development, and the risk of metabolic diseases in offspring. Carbohydrates play a crucial and multifaced role in regulating glucose and lipid metabolism in pregnant women and fetal growth. For expectant mothers in good health, a sufficient intake of high-quality carbohydrates is beneficial for both maternal and fetal health. Given that carbohydrates have a greater impact on postprandial blood sugar levels compared to other macronutrients, pregnant women with glucose metabolism disorders are advised to focus on improving the quality of their carbohydrate intake during pregnancy. It is imperative to conduct thorough research on the impact of carbohydrate diets during pregnancy on early fetal metabolic health. It is reasonable to hypothesize that increasing the proportion of high-quality carbohydrate consumption can lead to stable metabolic health during pregnancy and promote optimal offspring development. 

Carbohydrates play a complex and critical role in maintaining the homeostasis of glucose and lipid metabolism in pregnant mothers and fetal growth and development. Since carbohydrates increase postprandial blood sugar more than other macronutrients, pregnant women with glucose metabolism disorders are advised to improve the quality of their carbohydrate intake during pregnancy. A systematic study on the dietary intervention of carbohydrates during pregnancy will be of great significance to fetal metabolic health in early life. We can foresee that increasing the proportion of high-quality carbohydrates consumed is conducive to stable metabolic health during pregnancy and good development of offspring.

Future research on diet/nutrition during pregnancy considers the health and nutritional requirements of both the pregnant woman and the fetus. It is important that the quantity and quality of carbohydrate intake not only meet the nutritional needs of the fetus but also address the stable changes in blood sugar levels in pregnant women after meals. Special attention should be given to providing high-quality carbohydrates during pregnancy for women who are obese, have a history of diabetes, gestational diabetes, or high blood pressure. Further exploration into the impact of different carbohydrate foods on pregnancy health and offspring development is needed, followed by utilizing dietary pattern analysis to evaluate the correlation between carbohydrate intake, quality, and pregnancy outcomes.

## Figures and Tables

**Table 1:** Dietary carbohydrate and pregnancy complications.

Study	Geographic Area	Study Design	Participants	Periods	Dietary	Outcomes
Bao et al. (2016) [20]	USA	Ongoing prospective cohort study	4502 women with a history of GDM	1991–2011	Low-carbohydrate diets	Low-carbohydrate dietary patterns, especially those high in protein and fat from primarily animal foods, were associated with a higher risk of T2DM, while low-carb dietary patterns high in protein and fat from plant-based foods were not significantly associated with T2DM.
Rayner et al. (2020) [21]	Australian	Australian longitudinal study on women’s health cohort	9689 women	2001 (aged 50–55) and 2013 (aged 62–67)	Low-carbohydrate diets	Carbohydrate restriction was associated with a higher incidence of T2DM in middle-aged women, independent of a history of GDM.
Pang et al. (2017) [22]	Singapore	A birth cohort study	1247 pregnant women	18 and 46 y from 2009 to 2010	Low-carbohydrate diets	Higher intake levels of both animal and vegetable protein were associated with a higher risk of GDM in Asian women.
Saldana et al. (2004) [23]	USA	Prospective cohort study	1698 women in pregnancy	1 August 1995–31 May 2000	Adding 100 kcal from carbohydrates/substituting fat for carbohydrates	Adding 100 kcal of carbohydrates to the diet was associated with a lower risk of impaired glucose tolerance and a 9% lower risk of GDM; Replacing carbohydrates with fat resulted in a significantly increased risk of GDM.
Renault et al. (2015) [24]	Denmark	Treatment of obese pregnant women study	425 women at 11–14 weeks gestation	April 2009 to March 2012	/	Pregnant women who are obese can limit pregnancy weight gain by reducing their intake of sweets, snacks, and soft drinks.
Schoenaker et al. (2016) [25]	Australia	An ongoing population-based cohort study	9081 women aged 18–23 y	2003–2012	Mediterranean diet	Interventions that successfully implement a Mediterranean diet before pregnancy could significantly reduce the risk of GDM and HDP by optimizing preconception BMI.
Zhang et al. (2021) [26]	China	A prospective cohort study	10,126 women	2014–2017	/	Dietary GI, GL, and fiber intake influenced glucose homeostasis in Chinese pregnant women before and during pregnancy.
Hinkle et al. (2019) [27]	Denmark	A prospective cohort study	1274 pregnant women with GDM	2012–2014	Consumption of artificially sweetened beverages	Consumption of artificially sweetened beverages during pregnancy was associated with higher glycated hemoglobin (HbA1c), insulin, HOMA-IR, triglycerides, liver fat and obesity, and lower HDL at follow-up.
Hillesund et al. (2014) [28]	Norway	A prospective, population-based, pregnancy cohort study	66,597 women	1999–2008	High in complex carbohydrates	High consumption of fruits, vegetables, whole grains, potatoes, fish, milk, and drinking water during pregnancy may help normal-weight women achieve optimal pregnancy weight gain.
Crowther et al. (2018) [29]	New Zealand	A multicenter, stepped wedge, cluster, randomized trial	1080 pregnant women with GDM	/	/	More rigorous glycemic control in women with GDM resulted in a reduction in maternal and perinatal adverse outcomes.
Hu et al. (2014) [30]	China		140 pregnant women with GDM	/	Low GI staple diet	A low GI staple diet significantly reduced postprandial glucose levels in women with GDM.
Wrottesley et al. (2017) [31]	South Africa	A large longitudinal pregnancy cohort study	1000 pregnant women	/	/	Increasing intake of whole grains, legumes, vegetables, and traditional meats, and reducing intake of refined, high-sugar, and high-fat diets, may reduce the risk of excessive weight gain.
Skrede et al. (2018) [32]	Norway	The Norwegian mother and child cohort study	55,056 women	1999–2008	High in complex carbohydrates	Women whose dietary scores tended to include Nordic fruits, root vegetables, cabbage, potatoes, oatmeal, whole grains, wild fish, game, berries, milk, and water during pregnancy had lower average BMIs and less weight gain at 8 years postpartum, benefiting from long-term weight regulation.

**Table 2:** Dietary carbohydrate and offspring health outcomes.

Study	Geographic Area	Study Design	Participants/Model	Dietary	Outcomes
Kosiek et al. (2022) [106]	/	/	30 2-month-old female Wistar rats	Ketogenic diet during gestation and lactation	The ketogenic diet reduced the weight of the offspring and impairs the physical and neurological development of their offspring. Stopping this diet early postpartum may initiate the compensatory process and considerable recovery of nervous system function.
Viana et al. (2014) [107]	USA	Systemic reviews and meta-analyses	/	Low-GI diets	A low GI diet was associated with less insulin use and lower birth weight and was the most appropriate dietary intervention for patients with GDM.
Crowther et al. (2018) [29]	New Zealand	A multicenter, stepped wedge, cluster, randomized trial	1080 pregnant women with GDM	/	Stricter glycemic control in women with GDM significantly reduced large gestational age infants.
Sussman et al. (2013) [108]	/	/	Female mice were fed a standard diet and a ketogenic diet before and during pregnancy	Ketogenic diet (0.6% carbohydrate, 67.4% fat, 15.3% protein)	Prenatal and early postnatal exposure to the ketogenic diet caused significant changes in the brain structure of newborns and led to physiological developmental delays. These changes may be accompanied by functional and behavioral changes in later postpartum life.
Yisahak et al. (2021) [109]	USA	Diverse multisite cohort	1948 pregnant women	Healthy diet with high-quality carbohydrates	High-quality carbohydrate diet before and during the first trimester was beneficial for fetal growth.
Peraita et al. (2018) [110]	Spain	Two-phase retrospective population-based study	1118 pregnant women	High-quality carbohydrate diet	A high-quality maternal diet was associated with a lower risk of newborn birth and a reduced risk of preterm birth.
Zulyniak et al. (2020) [103]	Canada	Consortium of prospective cohort	2160 mother–infant pairs	Plant-based diet	A plant-based diet during pregnancy was associated with a lower incidence of infant eczema 1 year later.
Botto et al. (2016) [104]	USA	Multicenter population-based case–control study	19,353 mothers	Diet with high carbohydrate quality	Better maternal diet quality (six components were positively scored: legumes, whole grains, fruits and nuts, vegetables, fish, and the ratio of monounsaturated to saturated fatty acid intake) was associated with a reduced occurrence of some conotruncal and septal heart defects.
Zhu et al. (2017) [111]	Denmark	A longitudinal cohort	918 mother-singleton child dyads	High refined-grain intake	Higher maternal refined-grain intake during pregnancy was significantly related to a greater body mass index and a higher risk of overweight or obesity at age 7 y among children.
Helena et al. (2022) [112]	/	/	23 female Wistar rats with 10-week-old	High-sugar, high fat diet	Maternal HFS diet increased body weight, visceral fat, and serum total cholesterol, triglyceride, and leptin levels in weaned male rats, and decreased hypothalamic weight and increased adult visceral fat.
Sussman et al. (2013) [108]	/	/	Six-week-old female mice	Extremely low-carbohydrate diet (0.6% carbohydrate, 67.4% fat, 15.3% protein)	Prenatal and early postnatal exposure to the ketogenic diet caused significant changes in the brain structure of newborns and led to physiological developmental delays. These changes may be accompanied by functional and behavioral changes in later postpartum life.
Blumfield et al. (2015) [113]	Australia	Prospective, longitudinal cohort	129 mother–child dyads	Lower protein-to-carbohydrate ratio diet	The ratio of protein to carbohydrate intake during pregnancy was associated with the 4-year systolic blood pressure trajectory of children, and the systolic blood pressure of children was greatest during pregnancy when the proportion of high carbohydrate intake was high.
Nettleton et al. (2020) [114]	/	/	Female Sprague Dawley rats	Low-dose aspartame and stevia consumption with an obesogenic diet	Maternal consumption of high-calorie sweeteners during pregnancy disrupted weight regulation, glucose control, and gut microbiota early in life in both mothers and offspring.
Sideratou et al. (2018) [115]	/	/	C57BL/6 female mice	High GI diets	Mothers fed a high GI diet had higher expression of obesity-related genes in the placenta, and their offspring had 2.5 times higher expression of obesity-related genes in the hypothalamus at 20 weeks of age. The maternal rats’ high GI dietary carbohydrates during pregnancy are digested and absorbed more rapidly, reprogramming the offspring’s appetite.
